# Inhibition of murine herpesvirus-68 replication by IFN-gamma in macrophages is counteracted by the induction of SOCS1 expression

**DOI:** 10.1371/journal.ppat.1007202

**Published:** 2018-08-03

**Authors:** Yong Shen, Saisai Wang, Fangfang Sun, Gang Zheng, Tingting Wu, Yushen Du, Suzhan Zhang, Jing Qian, Ren Sun

**Affiliations:** 1 Cancer Institute (Key Laboratory of Cancer Prevention and Intervention, China National Ministry of Education, Key Laboratory of Molecular Biology in Medical Sciences, Zhejiang Province, China), The Second Affiliated Hospital, School of Medicine, Zhejiang University, Hangzhou, P. R. China; 2 Research Center of Infection and Immunity, ZJU-UCLA Joint Center for Medical Education and Research, Collaborative Innovation Center for Diagnosis and Treatment of Infectious Diseases, Zhejiang University School of Medicine, Hangzhou, P. R. China; 3 Department of Cardiology, the Second Affiliated Hospital, Zhejiang University School of Medicine, Hangzhou, P. R. China; 4 Department of Molecular and Medical Pharmacology, David Geffen School of Medicine, University of California at Los Angeles, Los Angeles, California, United States of America; 5 Pharmaceutical Informatics Institute, College of Pharmaceutical Sciences, Zhejiang University, Hangzhou, P. R. China; Stony Brook University, UNITED STATES

## Abstract

Gamma interferon (IFN-γ) is known to negatively regulate murine gammaherpesvirus-68 (MHV-68 or γHV-68) replication. This process involves the suppression of the viral gene replication and transcription activator (RTA) promoter, as well as activation of signal transducers and activators of transcription (STAT1). Notably, this effect is gradually attenuated during MHV-68 infection of bone marrow-derived macrophages (BMMs), which raised the possibility that the virus may utilize a mechanism that counteracts the antiviral effect of IFN-γ. By identifying the cellular factors that negatively regulate JAK-STAT1 signaling, we revealed that the infection of BMMs by MHV-68 induces the expression of suppressor of cytokine signaling 1 (SOCS1) and that depletion of SOCS1 restores the inhibitory effect of IFN-γ on virus replication. Moreover, we demonstrated that the expression of SOCS1 was induced as a result of the Toll-like receptor 3 (TLR3) mediated activation of the NF-κB signaling cascade. In conclusion, we report that TLR3-TRAF-NF-κB signaling pathway play a role in the induction of SOCS1 that counteracts the antiviral effect of IFN-γ during MHV-68 infection. This process is cell type-specific: it is functional in macrophages, but not in epithelial cells or fibroblasts. Our study reveals a mechanism that balances the immune responses and the escape of a gamma-herpesvirus in some antigen-presenting cells.

## Author summary

While viruses have developed various mechanisms to evade immune responses, hosts also have mechanisms to negatively regulate the antiviral signaling pathways to minimize potential damage. In this study, we show that MHV-68, a gamma-herpesvirus, is able to stimulate macrophages to produce the cellular protein SOCS1, which reduces the antiviral effect initiated by IFN-γ, in a cell type specific manner. These findings provide one more example to support the concept that viruses utilize SOCS1 as an immune evasion mechanism. We also show that TLR3-NF-κB signaling is responsible for the induced production of SOCS1. Our finding that TLR3/NF-κB/SOCS1 impedes the action of IFN-γ/STAT1 on RTA might offer a reasonable explanation of how virus-host interaction achieves a balance to facilitate intra-host spreading and transmission.

## Introduction

Murine gamma-herpesvirus 68 (MHV-68 or γHV-68) naturally infects rodents and is genetically and biologically related to two human gamma-herpesviruses, Epstein-Barr virus (EBV) and Kaposi's sarcoma-associated herpesvirus (KSHV) [1,2]. Like all other herpesviruses, MHV-68 has two distinct life-cycle phases: lytic replication and latency. Intranasal infection of MHV-68 in laboratory mice results in a productive infection in lung epithelial cells and latency in B lymphocytes, dendritic cells, and macrophages [3–7]. The lytic replication of MHV-68 is characterized by the sequential expression of immediate-early, early, and late viral genes [8]. Replication and transcription activator (RTA) is an immediate-early gene product that is encoded primarily by open reading frame 50 (ORF50), which initiates the lytic gene expression program, and controls the switch from latency to lytic replication [9,10].

Gamma interferon (IFN-γ) is the sole member of type II interferon family [11]. Its antiviral activity against several herpesviruses has been demonstrated for human cytomegalovirus (HCMV), varicella-zoster virus (VZV), herpes simplex virus-1 (HSV-1), and HSV-2 [12–14]. With respect to MHV-68, initial studies have identified IFN-γ as a key regulator of the reactivation MHV-68 from latency[15–18]. In bone marrow-derived macrophages (BMMs), IFN-γ can inhibit the promoters of the MHV-68 lytic switch gene RTA via the signal transducers and activators of transcription 1 (STAT1), resulting in reduced expression of RTA and inhibited viral lytic replication [7]. Thus, the inhibitory effect of IFN-γ/STAT1 on MHV-68 RTA expression is recognized as an antiviral mechanism.

Binding of IFN-γ to its cell-surface receptor IFN-γ receptor (IFNGR) results in the activation of the JAK-STAT1 signaling pathway followed by the translocation of STAT1 to the nucleus to activate gene transcription. In general, in order to regulate gene expression, STAT1 binds to the IFN-γ-activation site (GAS) elements present in the IFN-γ-responsive promoters [11]. However, in MHV-68 infected primary macrophages, although IFN-γ represses the RTA promoters in a STAT1-dependent manner, it does so in a way that is independent of the predicted GAS elements in the RTA promoter [7]. Although several cellular proteins, including suppressors of cytokine signaling (SOCS) proteins [19] and protein tyrosine phosphatases (PTPs) [20], have been identified as negative regulators of the JAK/STAT signaling pathway, it has not been determined whether or not IFN-γ-induced STAT1 signaling is regulated during MHV-68 infection.

Toll-like receptors (TLRs) are a group of well-characterized pattern recognition receptors (PRRs) that recognize pathogen-associated molecular patterns (PAMPs) such as lipopolysaccharide, peptidoglycans, flagellin, lipoteichoic acid, and unmethylated CpG DNA from various pathogens [21]. Activation of TLR signaling initiates an innate immune response and results in the production of IFNs, cytokines [21], as well as the development of adaptive immunity [22]. MHV-68, as well as its related human gamma-herpesviruses KSHV, can be detected by several TLRs including TLR2, -3, -4, -7, and -9 [23–26]. Despite this, the interaction between individual TLRs and the virus produces different biological responses. For example, TLR9 [23] and TLR2 [25] are involved in the antiviral immunity; TLR7 and/or TLR9 and their downstream NF-κB pathway play a role in MHV-68 reactivation [27]; and viral RTA is able to inhibit TLR2 and TLR4 signaling in BMMs [26]. It has been reported that a lack of TLR3 does not affect MHV-68 lytic replication and latency establishment [28]. Nevertheless, there are also reports showing that pre-activation of TLR3 by polyinosinic-polycytidylic acid (poly(I:C)) inhibits MHV-68 replication in BMMs [29], and that activation of TLR3 leads to MHV-68 reactivation in mice [24]. Therefore, TLR3 may play roles in addition to its antiviral effect. The impact of TLRs, especially TLR3 on MHV-68 infection and immune responses remains to be further clarified.

In the present study, we aimed to define the virus-inducible cellular factors that regulate the JAK/STAT signaling pathway and counteract the antiviral effect of IFN-γ. We identified SOCS1 as one such cellular factor. When triggered by MHV-68 infection, the TLR3 downstream NF-κB signaling pathway induces SOCS1 expression. Thus, we have discovered a novel mechanism that limits the impact of IFN-γ, which provides an escape for the virus, but at a controlled level (so called ‘controlled viral escape’).

## Results

### Loss of the antiviral effect of IFN-γ during MHV-68 infection in BMMs

**Fig 1:**
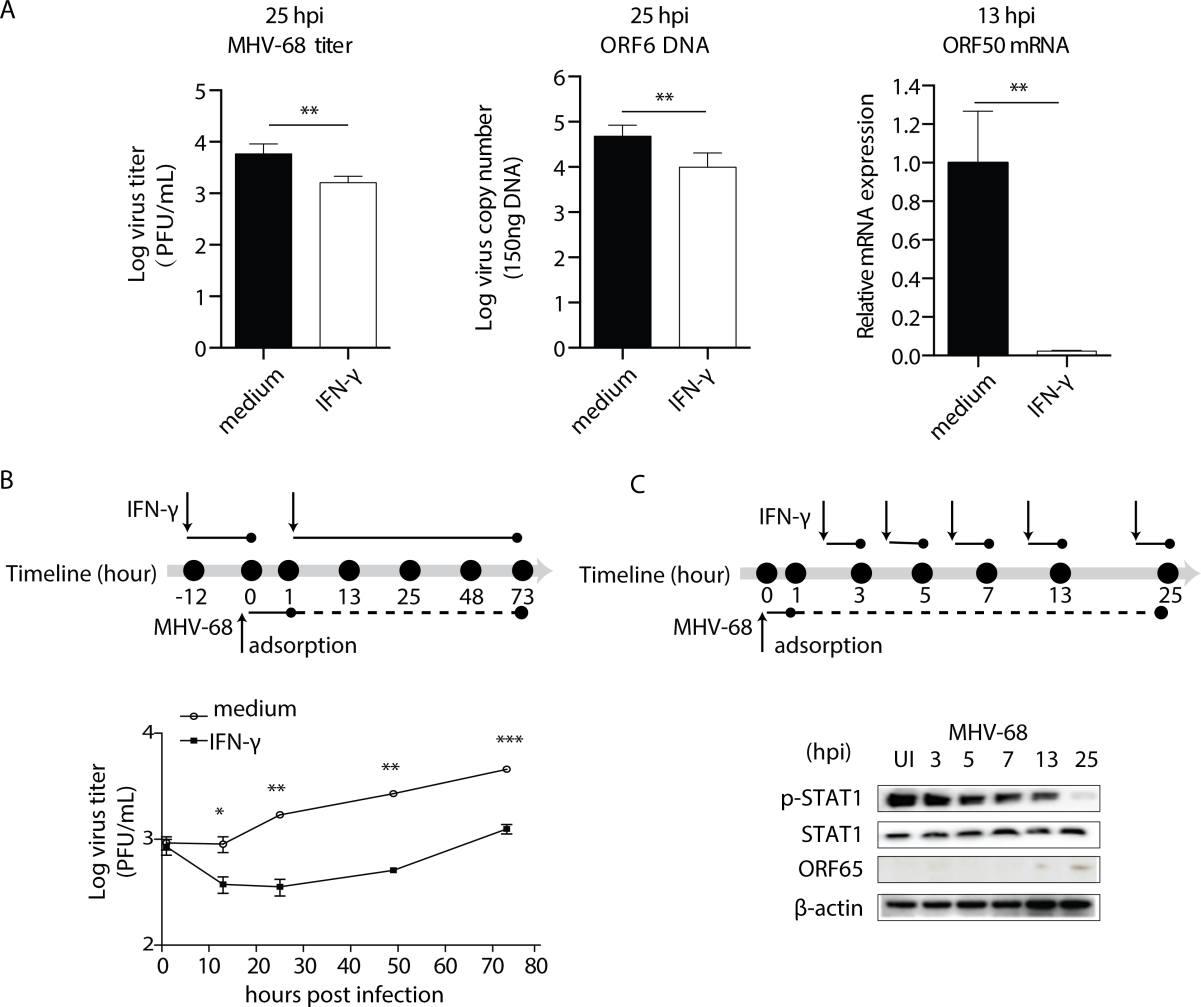
The gradual loss of the IFN-γ antiviral effect is accompanied by decreased STAT1 phosphorylation in BMMs. (A) BMMs were pre-treated with recombinant mouse IFN-γ at 10 U/mL, or medium for 12 hours before being infected with MHV-68 at MOI = 10. At the indicated hours post infection (hpi), cells were collected for plaque assay, ORF6 DNA PCR, and ORF50 RT-qPCR to determine virus titer, viral DNA copy number, and RTA expression, respectively. The ORF50 mRNA levels are expressed as values relative to the MHV-68 infected cells without IFN-γ treatment. (B) BMMs were pre-treated with recombinant mouse IFN-γ at 10 U/mL for 12 hours before being infected with MHV-68 at MOI = 10. At the indicated hours post infection, the virus titer was determined by plaque assay. The viral growth curve was created based on the Log virus titer (PFU/mL). (C) BMMs were infected with MHV-68 at MOI = 10 and allowed to grow for the indicated periods of time. At the beginning of the last hour in each time period, 10 U/mL of IFN-γ (w/IFN-γ) was added to the culture medium. At the end of each period, the cells were collected for protein extraction, and western blotting was performed to evaluate the expression levels of STAT1 tyrosine phosphorylation (p-STAT1) and STAT1. At the same times, ORF65 western blotting was performed to measure MHV-68 protein expression levels. The results are representative of three independent experiments and are expressed as means ± S.E.M. *p < 0.05, **p < 0.01, ***p < 0.001.

IFN-γ has a negative regulatory effect on MHV-68 lytic replication in BMMs and that this inhibition is mediated via STAT1 [7]. In our first set of experiments, we assessed the growth pattern of MHV-68 in the presence and absence of IFN-γ. As shown in Fig 1A, treatment with IFN-γ resulted in a reduced number of mature virus particles at 25 hours post-infection (hpi) as measured by CFU and ORF6 DNA copy number, as well as reduced ORF50 expression at 13 hpi. The growth curve showed that the treatment of BMMs with IFN-γ on BMMs significantly inhibited the MHV-68 replication (Fig 1B). These results are in accordance with Goodwin’s report [7]. Interestingly, we noticed that while the viral titer remained at a low level in the first day after IFN-γ treatment, the virus started to grow again since the viral tier was seen to increase at later times (Fig 1B).

The inhibition of viral replication by IFN-γ relies largely on the activation of the JAK-STAT1 signaling pathway [7]. In the next set of experiments, we collected BMMs at 3, 5, 7, 13, and 25 hpi respectively and monitored their response to IFN-γ. Cells were exposed to IFN-γ for one-hour before their phosphorylation status of STAT1 was determined by western blotting. As shown in Fig 1C, infection with MHV-68 alone did not induce phosphorylation of STAT1, whereas treatment with IFN-γ did. However, the IFN-γ-induced phosphorylation of STAT1 gradually declined over the 25-hr period in the infected BMM cells without any change in the total STAT1 protein level. This gradual decline in the level of STAT1 phosphorylation was not due to an endogenous feedback regulatory loop modulating IFN-γ signaling over the time course of the experiments, since mock-infected cells or cells infected with UV-inactivated MHV-68 showed sustained p-STAT1 expressions (S1 Fig). Such a reduction in STAT1 phosphorylation correlated with the decreased antiviral response induced by IFN-γ. Therefore, we speculated that the gradual loss of the IFN-γ antiviral effect in BMMs is due to the reduction in STAT1 phosphorylation that occurs during MHV-68 infection.

### MHV-68 infection induces SOCS1 expression in BMMs

**Fig 2:**
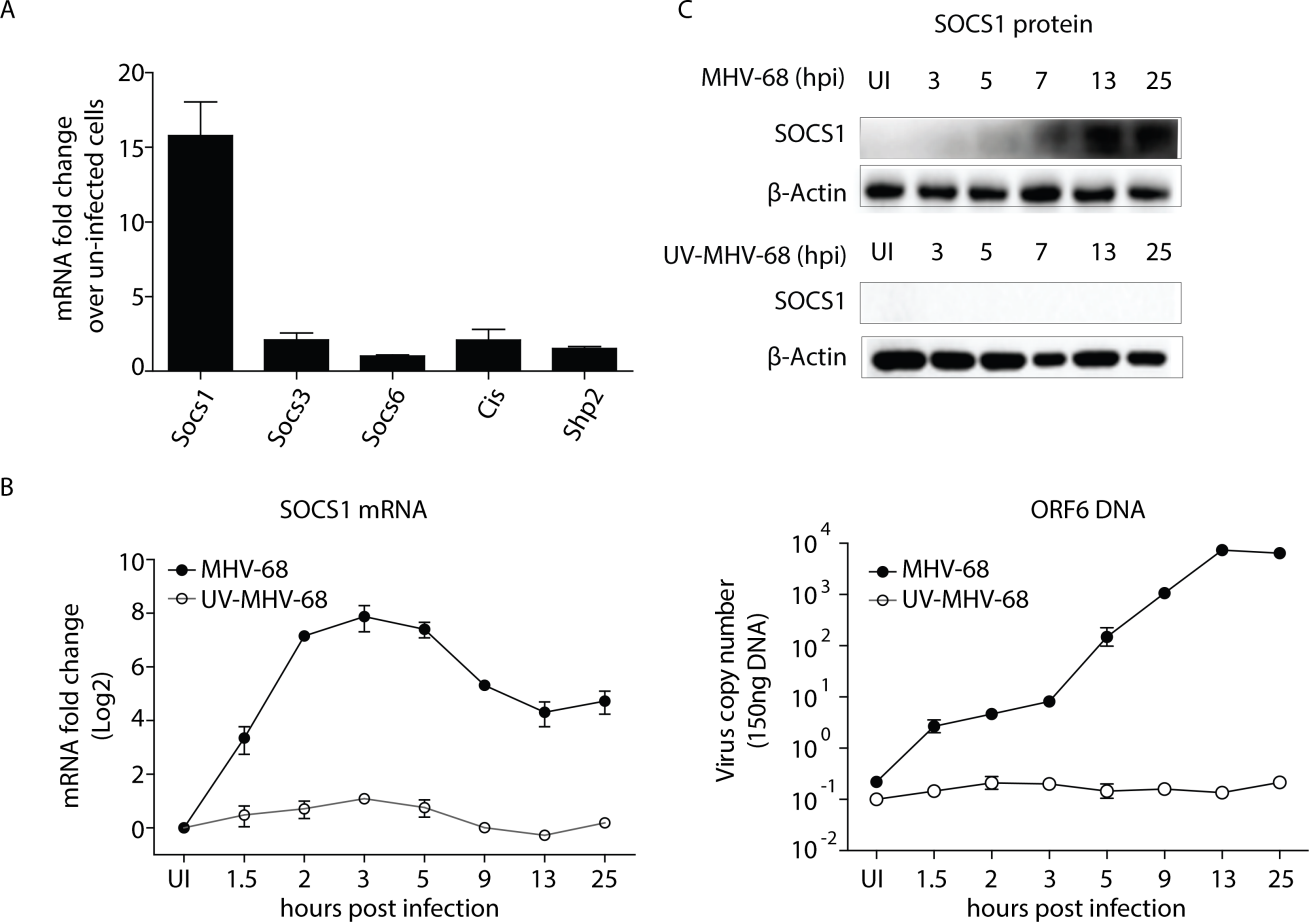
MHV-68 induces SOCS1 expression in BMMs. (A) BMMs were infected with MHV-68 at MOI = 10. At 13 hpi, total RNA was extracted and the mRNAs levels of Socs1, Socs3, Socs6, Cis and Shp2 were determined by RT-qPCR. (B-C) BMMs were either infected with MHV-68 or UV-inactivated MHV-68. At the indicated hours post infection, the cells were collected for RT-qPCR and western blotting to evaluate the SOCS1 mRNA and protein expression levels. In addition, ORF6 DNA qPCR was performed to assess the extent of viral replication. SOCS1 qPCR data are expressed as fold change in mRNA level compared to that in uninfected cells. The results are representative of three independent experiments and are expressed as means ± S.E.M.

Given that the phosphorylation of STAT1 can be negatively regulated by suppressors of cytokine signaling (SOCSs) and protein tyrosine phosphatases (PTPs) [30], we examined their expression in BMMs with and without MHV-68 infection. As shown in Fig 2A, among SOCS1, SOCS3, SOCS6, CIS and SHP2, SOCS1 exhibited a dramatic 16-fold induction in mRNA level. We next conducted an analysis of the SOCS1 expression kinetics during MHV-68 infection. An RT-qPCR analysis showed that the SOCS1 transcript was rapidly induced following MHV-68 infection, peaking at 2–4 hours after infection, and being continuously expressed until around 25 hours (Fig 2B). Consistent with the transcript levels, the level of the SOCS1 protein accumulated from 5- to 25 hpi in the infected BMMs (Fig 2C). Furthermore, incubation with UV-inactivated MHV-68 did not induce a detectable change in SOCS1 expression, implying that viral transcription is required to induce SOCS1 expression (Fig 2C).

### Inhibition of SOCS1 restores the antiviral effect of IFN-γ during MHV-68 infection in BMMs

**Fig 3:**
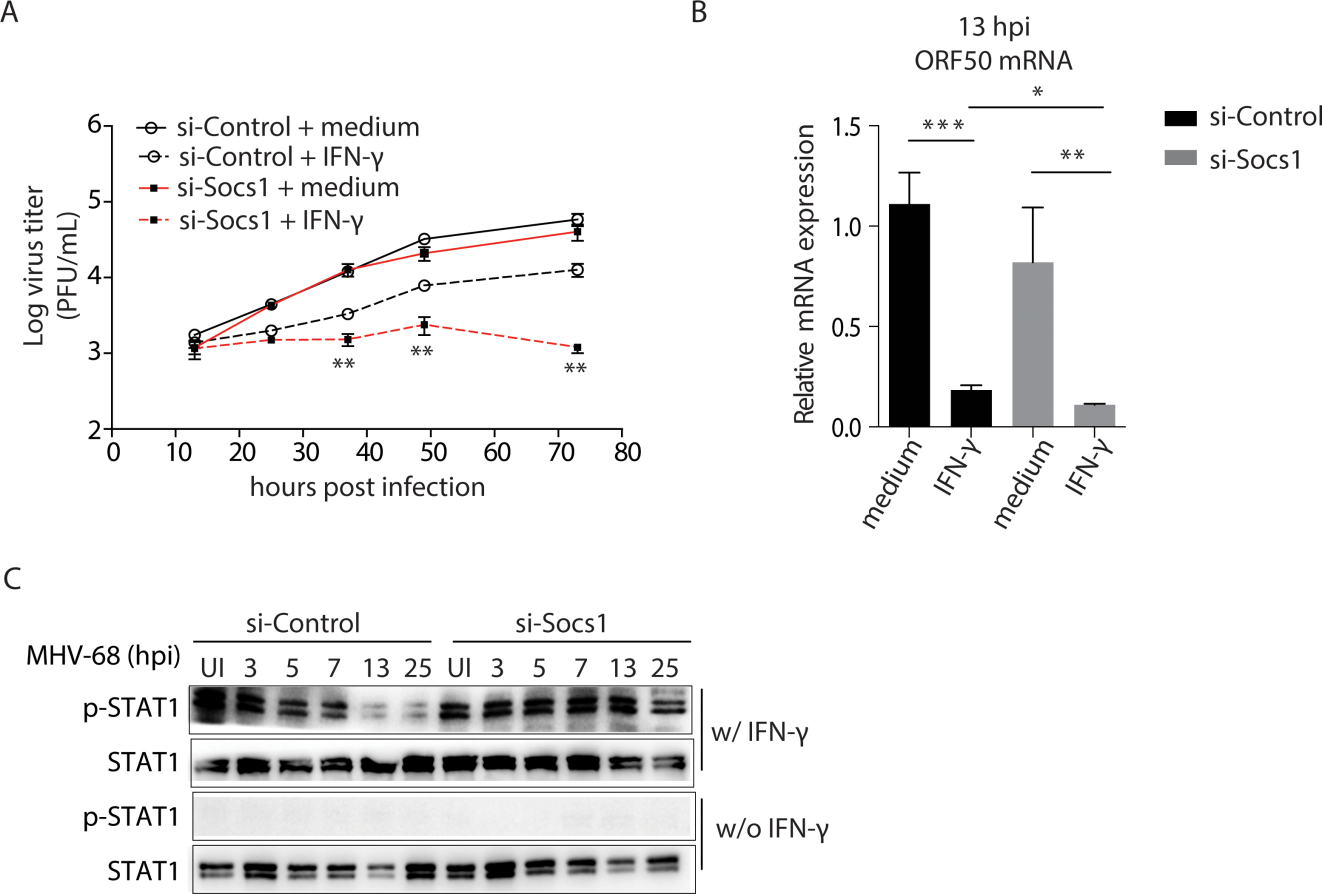
Knock down of SOCS1 results in a persistent antiviral effect of IFN-γ and tyrosine phosphorylation of STAT1 during MHV-68 infection. BMM cells were transfected with an siRNA against SOCS1 (si-Socs1) or an irrelevant target (si-Control). (A) At 24 hours after transfection, the cells were pre-treated with IFN-γ (10 U/mL) for 12 hours, and were then infected with MHV-68 at MOI = 10. At the indicated times post infection, the viral titer was measured by plaque assay. **p < 0.01, si-Socs1+IFN-γ *vs*. si-Control+IFN-γ. (B) At 13 hpi, viral ORF50 mRNA expression was detected by RT-qPCR. Data are expressed as relative values to the MHV-68 infected, si-Control transfected cells without IFN-γ treatment. *p < 0.05, **p < 0.01, ***p < 0.001. (c) Cells were infected with MHV-68 at MOI = 10 and allowed to grow for the indicated time periods. At the beginning of the last hour in each time period, 10 U/mL of IFN-γ (w/IFN-γ) or medium (w/o IFN-γ) was added to the culture medium. At the end of each time period, western blotting was performed to evaluate the expression levels of STAT1 tyrosine phosphorylation (p-STAT1) and STAT1. Results are representative of three independent experiments and are expressed as mean ± S.E.M.

To determine whether the induction of SOCS1 is responsible for the inhibition of IFN-γ activity, we knocked down SOCS1 expression using an siRNA approach, verifying the knockdown of SOCS1 by western blotting. Reducing SOCS1 expression levels had no effect on viral growth in the absence of IFN-γ; however, it restored the antiviral effect of IFN-γ during MHV-68 infection, and consequently, viral growth was continually inhibited by IFN-γ throughout the entire time course (see Fig 3A, at 36, 48, and 72 hpi, the differences between si-SOCS1 and si-Control cells treated with IFN-γ were statistically significant, *p<*0.01).

IFN-γ responsive inhibition of RTA can be detected at 13 hpi, earlier than changes in viral titer (Fig 1A and 1B and reference [7]). Therefore, we assessed this effect with and without knock down of SOCS1. A reduction in the expression level of SOCS1 itself did not interfere with RTA expression in MHV-68 infected BMMs. Nevertheless, the expression of RTA was inhibited by IFN-γ, and the absence of SOCS1 resulted in a further reduction in RTA expression (Fig 3B).

We also analyzed the levels of p-STAT1 status by western blotting in an experimental setting similar to that described for Fig 1C, albeit with and without knock down of SOCS1. Following treatment with IFN-γ, a decrease in STAT1 phosphorylation was observed in the control MHV-68 infected BMMs, consistent with our original findings. Knockdown of SOCS1 restored STAT1 phosphorylation in MHV-68 infected BMMs (Fig 3C). Taken together, we conclude that the induction of SOCS1 by MHV-68 infection primarily contributes to the inhibition of IFN-γ activity during MHV-68 infection in BMMs.

### Cell-type specific induction of SOCS1 in MHV-68 infected cells

**Fig 4:**
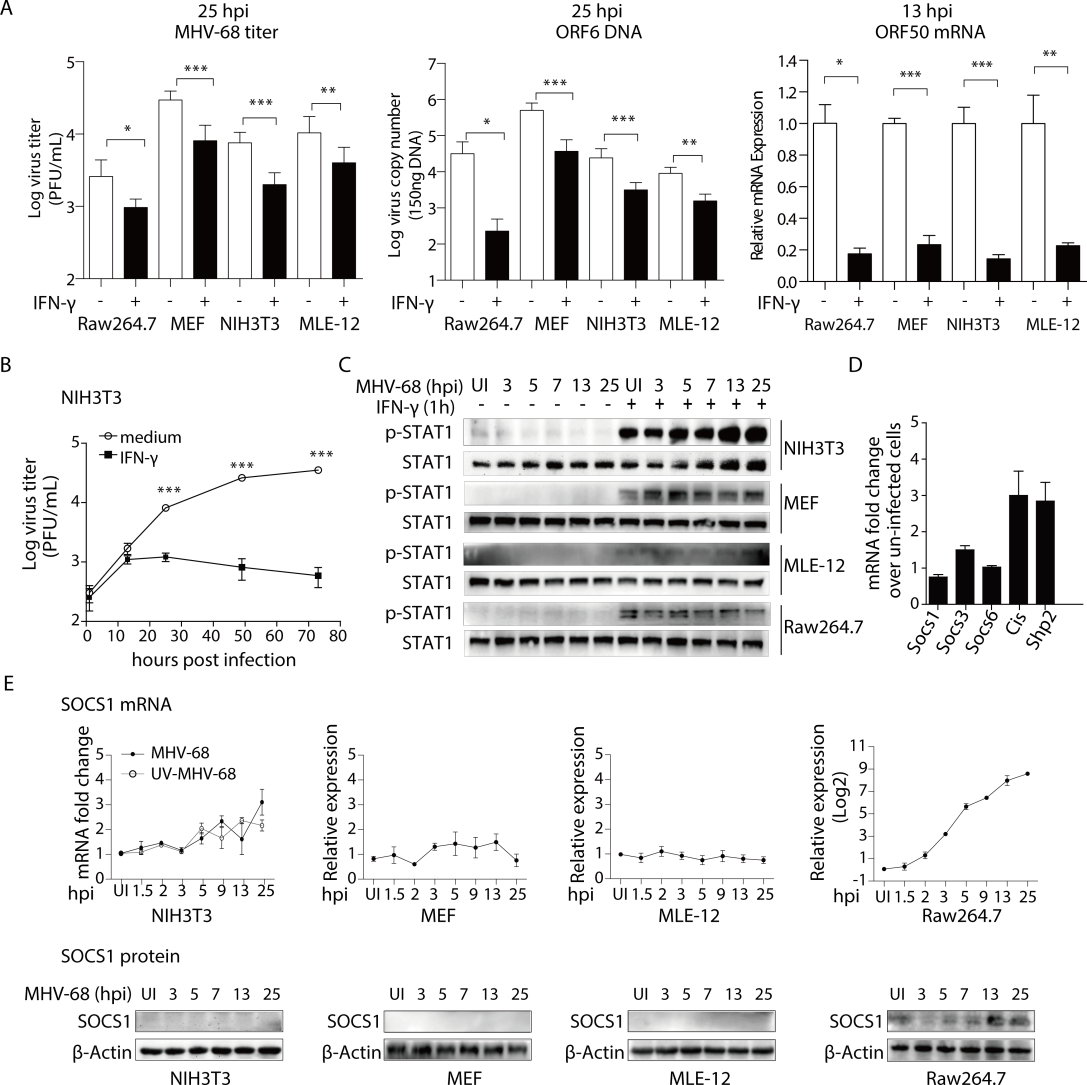
In NIH3T3 fibroblasts, MHV-68 infection does not lead to SOCS1 expression. (A) IFN-γ inhibits the lytic replication of MHV-68 in Raw264.7, MEF, NIH3T3, and MLE-12 cells. Raw264.7, MEF, NIH3T3, and MLE-12 cells were pre-treated with IFN-γ at 10 U/mL for 12 hours, and were then infected with MHV-68 at MOI = 10 (Raw264.7) or 1 (MEF, NIH3T3, MLE-12). At the indicated hours post infection (hpi), the cells were collected for plaque assay, ORF6 DNA PCR, and ORF50 RT-qPCR to evaluate virus titer, viral DNA copy number, and RTA expression, respectively. The ORF50 mRNA levels are expressed as values relative to the MHV-68 infected cells without IFN-γ treatment. (B) The antiviral role of IFN-γ is stable in NIH3T3 cells. NIH3T3 cells were pre-treated with IFN-γ at 10 U/mL for 12 hours before being infected with MHV-68 at MOI = 10. At the indicated hours post infection, the virus titer was determined by plaque assay. The viral growth curve was created based on Log virus titer (PFU/mL). (C) IFN-γ induced tyrosine phosphorylation of STAT1 is stable in NIH3T3, MEF, and MLE-12 cells, but gradually declines in Raw264.7 cells. NIH3T3, MEF, MLE-12, and Raw264.7 cells were infected with MHV-68 and allowed to grow for the indicated periods of time. At the beginning of last hour in each time period, 10 U/mL of IFN-γ was added to the culture medium. At the end of each time period, cells were collected for protein extraction, and western blotting was performed to evaluate the expression levels of p-STAT1 and STAT1. (D) MHV-68 does not induce Socs1, Socs3, Socs6, Cis, or Shp2 mRNA expression in NIH3T3 cells. NIH3T3 cells were infected with MHV-68 at MOI = 1. At 13 hpi, total RNA was extracted and the mRNAs of Socs1, Socs3, Socs6, Cis, and Shp2 were detected by RT-qPCR. (E) MHV-68 does not induce SOCS1 mRNA or protein expression in NIH3T3, MEF, and MLE-12 cells but induces both SOCS1 mRNA and protein expressions in Raw264.7 cells. Cells were either infected with MHV-68 or UV-inactivated MHV-68. At the indicated hours post infection, cells were collected for RT-qPCR and western blotting to evaluate SOCS1 mRNA and protein expression. qPCR data are expressed as fold change in mRNA level compared to that in uninfected cells. Results are representative of three independent experiments and are expressed as mean ± S.E.M., *p < 0.05, **p < 0.01, ***p < 0.001.

Next, we asked whether the inhibitory effect of IFN-γ and induction of SOCS1 expression occur in all MHV-68 infected cells or if it is restricted to certain cell types. In this regard, we first assessed the antiviral effect of IFN-γ in several types of cells including fibroblasts (NIH3T3, MEF), epithelial cells (MLE-12), and macrophages (Raw264.7). As shown in Fig 4A, IFN-γ treatment reduced MHV-68 virion production and viral DNA replication at 25 hpi and inhibited the expression of RTA at 13 hpi in all cell types tested. These data indicate that epithelial cells and fibroblasts are as susceptible as macrophages to the suppressive effect of IFN-γ. Nevertheless, in NIH3T3 cells, we did not observe the gradual recovery of viral replication and the gradual decline in STAT1 phosphorylation as was seen in BMMs. Instead, the viral titer remained suppressed (Fig 4B). At 3, 5, 7, 13, and 25 hpi, the level of STAT1 phosphorylation remained constant in response to IFN-γ in NIH3T3, MEFs, and MEL-12 cells. However, in infected Raw264.7 cells, the phosphorylation of STAT1 gradually declined over the 25-hr period (Fig 4C). There was no detectable SOCS1 expression in NIH3T3 cells, with or without MHV-68 challenge (Fig 4D and 4E). Similar results to those seen in BMMs following infection were also seen in Raw264.7 cells compared to MEF and MLE-12 cells, in that MHV-68 infection induced the expression of SOCS1 in Raw264.7 cells but not in MEF and MLE-12 cells (Fig 4E).

**Fig 5:**
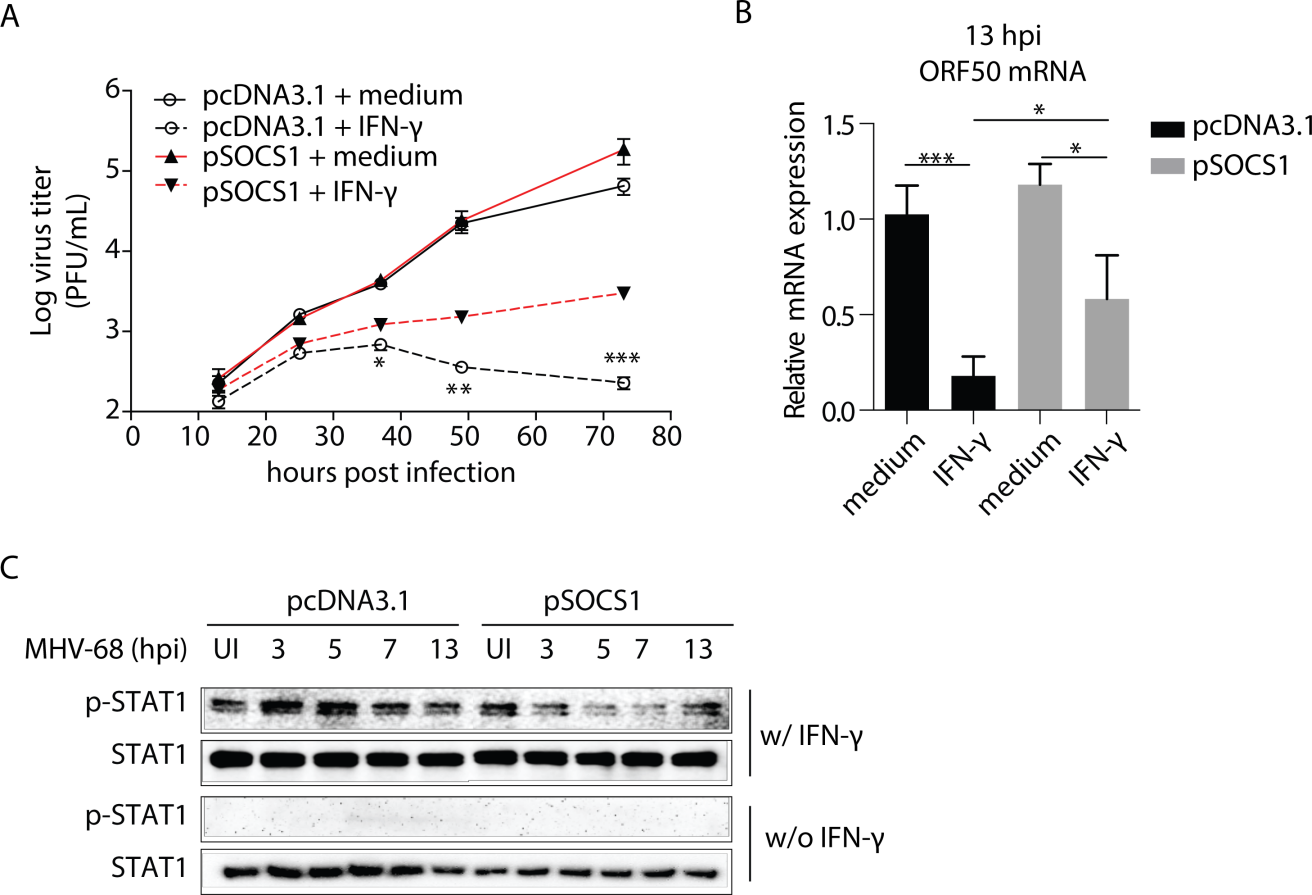
Overexpression of SOCS1 decreases the antiviral effect of IFN-γ and tyrosine phosphorylation of STAT1 during MHV-68 infection in NIH3T3 fibroblasts. NIH3T3 cells were transfected with an expression plasmid encoding SOCS1 (pSOCS1) or control (pcDNA3.1). (A) At 48 hours after transfection, the cells were pre-treated with IFN-γ (10 U/mL) for 12 hours, and were then infected with MHV-68 at MOI = 10. At the indicated times post infection, the viral titer was measured by plaque assay. *p < 0.05, **p < 0.01, ***p<0.001, pSOCS1+IFN-γ *vs*. pcDNA3.1+IFN-γ. In parallel, western blotting was performed to assess the SOCS1 expression levels in cells without IFN-γ treatment. (B) RT-qPCR was used to detect the viral ORF50 mRNA expression at 13 hpi. Data are expressed as values relative to the MHV-68 infected, pcDNA3.1 transfected cells without IFN-γ treatment. *p < 0.05, ***p < 0.001. (C) Cells were infected with MHV-68 for the indicated times, with the last hour of infection being in the presence of IFN-γ (10 U/mL) or not. Proteins were extracted, and western blotting was performed to evaluate the tyrosine phosphorylation level of STAT1. Results are representative of three independent experiments and are expressed as mean ± S.E.M.

When an SOCS1 expression plasmid was transfected into NIH3T3 cells, the induced expression of SOCS1 rendered the NIH3T3 cells resistant to IFN-γ similar to what was observed in BMMs. The growth curve analysis showed that inducing SOCS1 expression, while it had no effect on viral growth in the absence of IFN-γ, gradually diminished the antiviral effect of IFN-γ during MHV-68 infection, and consequently, the viral titer went up at 36, 48, and 72 hpi after IFN-γ treatment (*p<*0.05)(Fig 5A). At 13 hpi, while the expression of RTA was inhibited by IFN-γ, the presence of SOCS1 resulted in the restoration of RTA expression (*p<*0.05) (Fig 5B). In contrast to the consistent STAT1 phosphorylation levels observed in the control MHV-68 infected NIH3T3 cells, overexpression of SOCS1 resulted in a gradual loss of STAT1 phosphorylation following treatment with IFN-γ (Fig 5C).

### TLR3 mediates the MHV-68-induced SOCS1 production in BMMs

**Fig 6:**
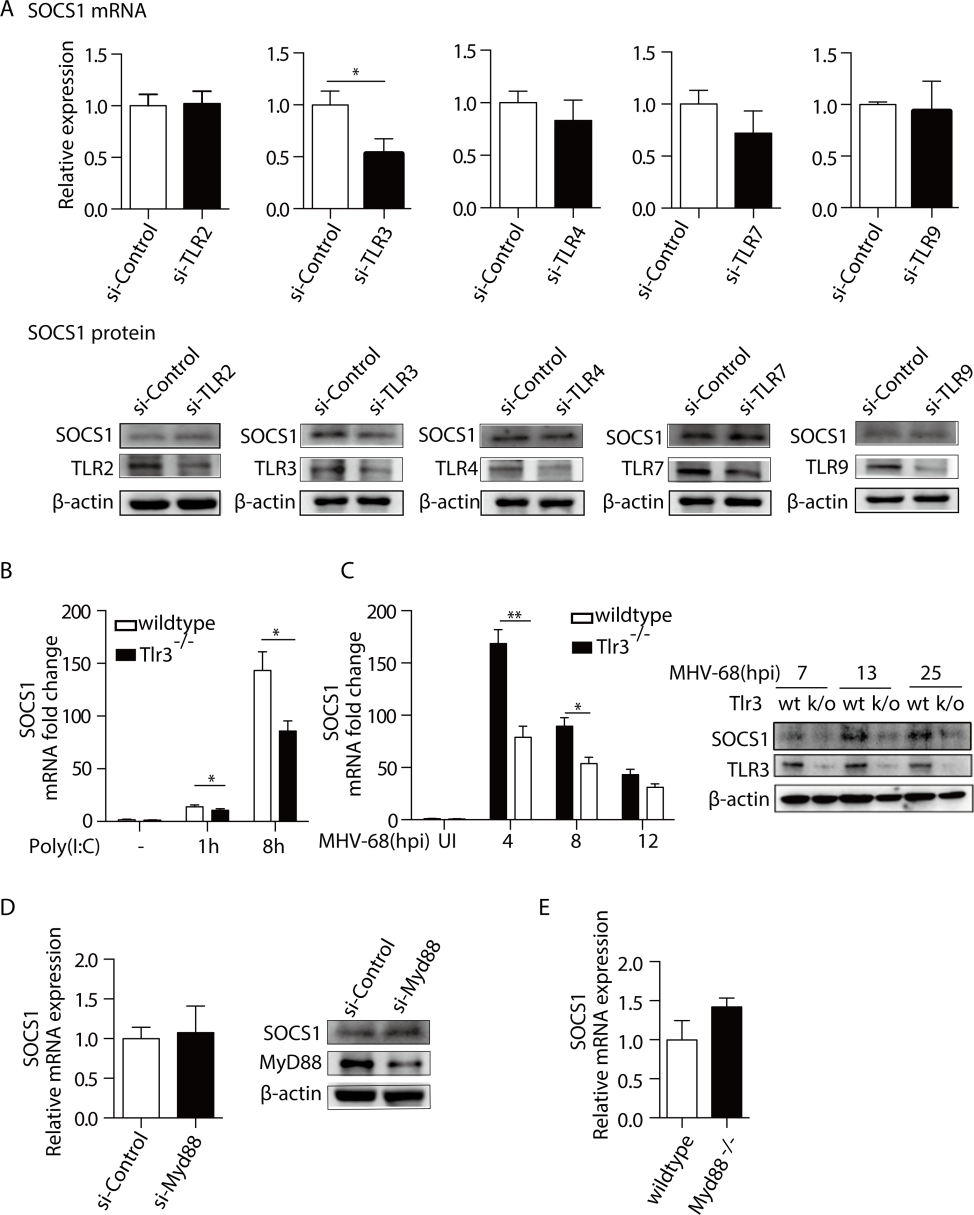
TLR3 mediates the MHV-68-induced SOCS1 production. (A) BMMs were transfected with siRNAs against Tlr2, Tlr3, Tlr4, Tlr7, Tlr9 (si-TLR) or an irrelevant target (si-Control), respectively. Forty-eight hours post transfection, cells were infected with MHV-68 at MOI = 10. At 8 hpi, RNA and protein were then extracted. RT-qPCR was performed to determine SOCS1 mRNA levels and western blotting was performed to determine the protein expression levels of SOCS1 and the individual TLRs, respectively. The SOCS1 mRNA levels are expressed as values relative to the MHV-68 infected, si-Control transfected cells. (B) BMMs from *Tlr3*^*-/-*^ and wildtype C57BL/6 mice were treated with poly(I:C) at 10 μg/mL for 1 and 8 hours, respectively. Total RNA was extracted and SOCS1 mRNA levels were determined by RT-qPCR. qPCR data are expressed as fold change in mRNA level compared to that in untreated WT cells. (C) BMMs from *Tlr3*^*-/-*^ mice and wild type (WT) mice were infected MHV-68. At indicated hpi, cells were harvested to measure the SOCS1 mRNA levels by the RT-qPCR, and to measure the TLR3 and SOCS1 proteins levels by western blotting. qPCR data were expressed as fold change in mRNA level compared to that in uninfected WT cells. (D) BMMs were transfected with si-MyD88 or si-Control. Forty-eight hours post-transfection, the cells were infected with MHV-68. At 8 hpi, the SOCS1 mRNA levels and protein levels were determined. The SOCS1 mRNA levels are expressed as values relative to the MHV-68 infected, si-Control transfected cells. (E) BMMs from *Myd88*^*-/*-^ mice and wildtype C57BL/6 mice were infected with MHV-68. At 8 hpi, total RNA was extracted and the mRNA expression of SOCS1 was determined by RT-qPCR. The SOCS1 mRNA levels are expressed as values relative to the MHV-68 infected WT cells. Results are representative of three independent experiments and are expressed as mean ± S.E.M., *p < 0.05.

Until now, the data suggested that the induction of SOCS1 expression during MHV-68 infection is a mechanism that can counteract the antiviral JAK-STAT1 pathway activated by IFN-γ. Next, we tried to uncover how MHV-68 infection induces SOCS1 expression. Based on the observation that immune cells and non-immune cells respond differently with respect to SOCS1 expression, and the knowledge that macrophages have high expression of Toll-like receptors (TLRs), (E-GEOD-63340 in Array Express), we hypothesized that certain TLR-specific signaling pathway control SOCS1 expression. With the established connection between TLRs (2, 3, 4, 7 and 9) and MHV-68 infection [23–26], we evaluated the impact of the silencing individual TLR on SOCS1 expression using an siRNAs approach (si-TLR2, 3, 4, 7, and 9, respectively). As shown in Fig 6A, knockdown of TLR3 significantly reduced MHV-68 stimulated SOCS1 expression, whereas knockdown of TLR2, TLR4, TLR7, and TLR9 had no effect. The knocking down efficiency for the individual siRNAs ranged from 50%-75%, as detected by western blotting (Fig 6A and S2 Fig).

Further experiments were performed to confirm that TLR3 regulates SOCS1 expression. Using BMMs derived from *Tlr3*^-/-^ or wild type (WT) mice, we treated BMMs with either poly(I:C), a known TLR3 agonist [31] or MHV-68 and checked the expression levels of SOCS1. As shown in Fig 6B, poly(I:C) significantly induced SOCS1 expression as its transcript level dramatically increased at 8 h after stimulation (*p <* 0.05), and this effect of which could be partially blocked in the absence of TLR3 (*p <* 0.05). TLR3 protein level was less than 10% in *Tlr3*^*-/-*^ BMM cells in comparison with the WT cells, as determined by western blotting (Fig 6C and S2 Fig). MHV-68 infection induced less SOCS1, at both the mRNA and protein levels in *Tlr3*^*-/-*^ BMMs compared to WT BMMs. Strong inhibition was seen at 4 hpi for SOCS1 mRNA (0.47 of WT levels) and 13 hpi for SOCS1 protein (0.46 of WT levels) (Fig 6C). In addition, we knocked down MyD88, a key signaling molecule that mediates TLRs signaling from TLRs other than TLR3, using either BMMs derived from *MyD88*^-/-^ mice or by si-RNA. As shown in Fig 6D and 6E, knockdown/knockout of MyD88 had no effect on the MHV-68 induced SOCS1 expression. Taken together, we conclude that TLR3 is a factor that promotes SOCS1 expression.

### NF-κB mediates the SOCS1 expression in BMMs upon MHV-68 infection

**Fig 7:**
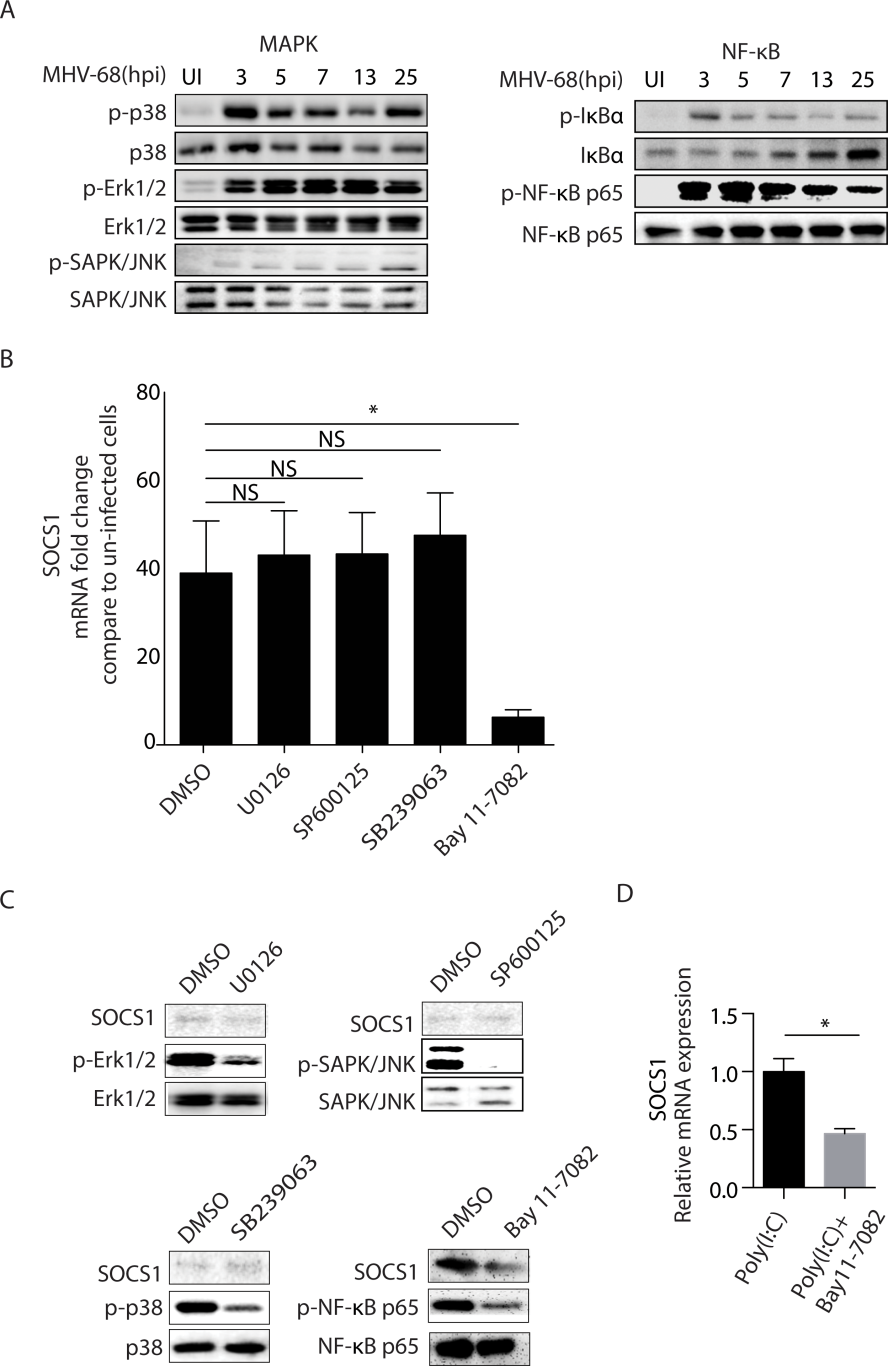
NF-κB is responsible for the MHV-68-induced SOCS1 production. (A) BMMs were infected with MHV-68 at MOI = 10. At 3, 5, 7, 13, and 25 hpi, protein was extracted and the levels of phospho-p38, p38, phospho-Erk1/2, Erk1/2, phospho-SAPK/JNK, SAPK/JNK, phospho-IκBα, IκBα, phospho-NF-κB p65, NF-κB p65 were determined by western blotting. (B) BMMs were pretreated with DMSO, U0126, SP600125, SB239063 or Bay11-7082, respectively for 30 min before being infected with MHV-68. At 8 hpi, SOCS1 mRNA was determined by RT-qPCR. Data are expressed as fold change in mRNA level compared to that in uninfected cells. (C) BMMs were pretreated with U0126, SP600125, SB239063, or Bay11-7082 for 30 min before being infected with MHV-68. At 8 hpi, the levels of SOCS1, phospho-NF-κB p65, NF-κB p65, phospho-Erk1/2, Erk1/2, phospho-p38, p38, phospho-SAPK/JNK, and SAPK/JNK were determined by western blotting. (D) BMM cells were pretreated with Bay11-7082 for 30 min before cells were stimulated with Poly(I:C) for 12 hours. Cells were collected for RNA extraction and SOCS1 expression was determined by RT-qPCR. Data are expressed as SOCS1 relative mRNA values over Poly(I:C) stimulated cells without inhibitor treatment. Results are representative of three independent experiments and are expressed as mean ± S.E.M., *p < 0.*05*.

We have shown that TLR3 is partially responsible for the increase in SOCS1 expression in BMM cells. We next wished to elucidate the downstream signaling pathway bridging TLR3 with SOCS1. In general, the activation of TLR3 induces TRIF adaptor dependent activation of NF-κB, mitogen-activated protein (MAP) kinases and phosphorylation of interferon regulatory factor 3 (IRF3), leading to the productions of type I IFNs as well as pro-inflammatory cytokines [32]. Potentially signaling through TLR3 (IRF-3, NF-κB and/or MAPK) and the production of resulting cytokines arising from TLR3 signaling, such as IFNs, could trigger the induction of SOCSs. Based on the fact that IFN-mediated SOCS induction via JAK/STAT1 occurs later and that MHV-68 encodes a kinase (ORF36) that can bind and inhibits the function of IRF3 [33], we decided to focus on the contribution of the NF-κB or MAPK signaling pathways to SOCS1 induction. First, we measured the phosphorylation of SAPK/JNK, p38, ERK1/2, IκBα and NF-κB p65, which represent the activation of the MAPK (JNK, p38, ERK) and NF-κB signaling pathways. Samples collected from 3, 5, 7, 13, and 25 hpi were examined, respectively. As shown in Fig 7A, MHV-68 infection led to the phosphorylation of p38, ERK, IκBα, and NF-κB p65; although weak, phosphorylation of JNK could also be detected. Second, we used a panel of chemical kinase inhibitors to further illuminate the relationship with between these kinase and SOCS1 expression during MHV-68 infection. Fig 7B and 7C show that the expression of SOCS1 was dependent on the activation of NF-κB since pre-incubation of cells with the NF-κB inhibitor Bay 11–7082 abolished the expression of SOCS1 induced by MHV-68 infection, whereas the JNK inhibitor SP600125, the p38 inhibitor SB239063 and the ERK inhibitor U0126 had no effect. Treatment with the individual inhibitors did not produce any detectable toxicity in the cells as measured by microscopy of cell morphology, as well as flow cytometry for annexinV/PI staining. Based on these findings, we conclude that MHV-68 transcription activates the signaling pathway leading from TLR3 to NF-κB to induce SOCS1 expression in BMMs.

## Discussion

In this report, we have shown that the IFN-γ-mediated inhibition of murine herpesvirus-68 replication is suppressed by TLR3-induced SOCS1 expression in macrophages. Specifically, MHV-68 induces the expression of SOCS1, a suppressor of cytokine signaling in the JAK-STAT1 pathway. This increased SOCS1 expression, induced by TLR3/ NF-κB signaling pathway, is functional in macrophages.

Viruses have evolved various mechanisms to counteract the host immune responses [34], either by a direct inhibitory effect of certain viral proteins or through the induced expression of negative regulators in infected cells [35–40]. Some of these inducible negative cellular factors have been identified for other members of the herpesvirus family. For instance, HCMV infection leads to the production of the tyrosine phosphatase Shp2, which inhibits IFN-γ-induced STAT1 tyrosine phosphorylation [41]. HSV-1 induces suppressor of cytokine signaling SOCS1 and SOCS3 expression in HEL-30 keratinocytes as well as in neuron-enriched trigeminal ganglia primary cultures, inhibiting the activity of IFN-β or IFN-γ [42]. Specifically with respect to MHV-68, a large number of studies have focused on elucidating the nature of viral proteins that can antagonize IFN production or antagonize the JAK-STAT1 signaling pathway that lies downstream of IFN. For instance, ORF11 reduces the interaction between TBK1 and IRF3 and subsequently inhibits activation of IRF3, thereby negatively regulating IFN-β production [43]. ORF36 suppresses IRF3-driven transcription of the IFN-β promoter as well as IFN-β production, by directly blocking the interaction between active IRF3 and the transcriptional co-activator CBP [44]. ORF54 efficiently inhibits type I interferon signaling by inducing degradation of the type I interferon receptor protein IFNAR1 [5]. ORF52 inhibits c-GAS, and thereby inhibits cGAS-dependent activation of IRF3 in cells [45]. In the current study, we identified SOCS1 as a cellular negative regulator of JAK-STAT1 activation downstream of IFN that is induced during MHV-68 infection.

Our study indicated that the virus-induced increase in SOCS1 expression in primary macrophages was mediated by TLR3 and NF-κB activation. Both TLRs [46] and NF-κB [47,48] have been reported to be regulators of SOCS1 expression. In addition to MHV-68 infection, we demonstrated that when TLR3 on BMMs was activated by poly(I:C), the SOCS1 expression was also induced (Fig 6B) in a manner dependent on NF-κB activation (Fig 7D). Thus, we demonstrated that the TLR3/NF-κB cascade is a signaling pathway that controls SOCS1 expression during MHV-68 infection. Notably, in *Tlr3*^*-/-*^ BMMs, MHV-68 infection was capable of inducing SOCS1 expression, but not to the same extent as in WT cells (Fig 6C), which suggests that TLR3 signaling is not the only mechanism by which the virus induces SOCS1. Other signaling pathways including IFN signaling pathways[49], the Hedgehog/GLI signaling pathway[50], the hypoxia activated HIF-1α pathway [51], and the virus-related STAT3 activation [52,53] might also participate in the process. In addition, innate immune pathways other than TLRs, such as cytosolic DNA sensing stimulator of IFN genes (STING) pathway [54], or the RNA sensing retinoic acid-inducible gene I (RIG-I) pathway [55], have been identified as regulators of MHV-68 replication and death. Whether or not these pathways play roles in regulating SOCS1 expression is worthy of future investigation.

TLR3 recognizes double-stranded viral RNAs, such as the overlapping transcripts generated during viral infection [56]. In the current study, we showed that UV-inactivated MHV-68 failed to induce a significant change in SOCS1 expression, but poly(I:C) successfully mimicked the effects of MHV-68. Our result is concordant with a previous study, which showed that only viable KSHV, but not UV-inactivated KSHV, could activate TLR3 signaling [57]. Therefore, when macrophages are infected with MHV-68 and treated with IFN-γ, the activation of the TLR3/NF-κB signaling pathway leads to the production of SOCS1. As a result, the virus can maintain its replication ability at a certain level.

It is apparent that macrophages respond differently, compared with epithelial cells and fibroblasts, to IFN-γ during MHV-68 infection. In epithelial cells and fibroblasts, there is no infection-induced expression of SOCS1, and thus virus replication was consistently reduced in response to IFN-γ. This cell type-specific expression of SOCS1 has been previously reported for other herpesviruses. For example, HSV-1 induces SOCS1 expression in HEL-30 keratinocytes but not in L929 fibroblasts [42], and in un-polarized (M0) but not in either M1- or M2-polarized murine J774A.1 macrophages [58]. Although not fully clarified, the induced expression of SOCS1 in HSV-1 infected keratinocytes is believed to contribute to its relative resistance to IFN therapy [42]. In addition, there is an early transient increase in SOCS1 mRNA expression in IC-21 mouse macrophages, but not in fibroblasts, following MCMV infection [59]. Our current study adds another player in this scenario, in the sense that MHV-68 induces SOCS1 expression in macrophages but not in epithelial cells or fibroblasts.

TLR3 plays important roles in both antiviral immunity and the induction of adaptive immunity. It is well established that TLR3 facilitates the maturation of dendritic cells (DCs) into potent immune-stimulatory cells endowed with the capacity to efficiently cross-prime T lymphocytes [54][60]. A similar role for TLR3 may also be applicable in macrophages, another powerful professional innate immune cell and antigen presenting cell [22][55]. Although the impact of TLR3 on the outcome of MHV-68 varies under different situations, it is likely that macrophages would require some degree of viral antigen production in order to process the virally derived peptides for presentation in the context of MHC-II. Therefore, our finding that TLR3/NF-κB/SOCS1 impedes the action of IFN-γ/STAT1 on RTA (the “controlled viral escape”) may be an important mechanism for promoting adaptive immunity to MHV-68.

MHV-68, like other herpesviruses, persists for a lifetime in the host, and lives between phases of latency and reactivation. Macrophages appear to be cells that are immediately infected upon transmission [61] and host both lytic and latent viruses *in vivo* [62,63]. Considering the fact that lytic replication plays a critical role in seeding the latent splenic reservoir [63,64], and that RTA [63,65,66], IFN-γ [67], and NF-κB [68,69] are important interacting players in MHV-68 viral latency and reactivation, our finding of a “controlled viral escape” might offer a reasonable explanation for how virus-host interactions, reach this balanced status, to facilitate viral intra-host spreading and transmission. The involvement of TLR3 in viral immune invasion has been previously reported. For example, the TLR3-TRIF pathway is responsible for KSHV RTA expression, and, KSHV RTA in turn degrades TRIF to block host innate immune responses[70]. Another related example is the West Nile virus, which is aided by TLR3 to gain entry into the central nervous system and induce lethal encephalitis [71].

In summary, we have identified a negative-regulatory mechanism acting on the IFN-γ pathway that is likely to be a part of the innate immune response for macrophages to promote adaptive immune responses or may be one of the MHV-68 evasion strategies to hinder the innate immune responses. This mechanism relies on the induced production of SOCS1, which exerts its inhibitory action on IFN-γ. This study should lead to further studies to define the roles for TLR3/SOCS1 in macrophages from the aspects of antiviral responses, inflammatory cytokine production, and antigen processing and to track the spread and fate of virus-infected macrophages *in vivo*.

## Materials and methods

### Viruses, cells and reagents

MHV-68 virus was originally obtained from the American Type Culture Collection (VR1465). Viral stocks of MHV-68 were obtained by infection of Vero cells (ATCC: CCL-81) at a multiplicity of infection (MOI) of 0.05. The titer of the viruses was determined using a plaque assay on Vero cells. Cells, including Vero, Raw264.7 (murine monocytic cell line, ATCC: TIB-71), NIH3T3 (mouse embryo fibroblast cell line, ATCC: CRL-6442) and MLE-12 (murine lung epithelial cells, ATCC: CRL-2110) were cultured in Dulbecco’s-modified Eagle’s medium (DMEM) supplemented with 10% heat-inactivated fetal bovine serum (FBS), 2 mM glutamine, 50 units/mL penicillin/streptomycin. Primary bone marrow macrophages (BMMs) were derived from wildtype C57BL/6 mice (6–8 week, female), *Tlr3*^*-/*-^ or *MyD88*^*-/*-^ mice (6–8 week, female) and were cultured in complete RPMI1640 with 10 ng/mL M-CSF for 7 days. Murine embryonic fibroblasts (MEFs) were obtained from C57BL/6 mouse embryos (14 days) and were cultured in complete DMEM medium. Recombinant murine IFN-γ and recombinant murine M-CSF were purchased from PeproTech (Rocky Hill, NJ, USA). All the cell culture reagents were purchased from Invitrogen (Shanghai, China). In some of the experiments, MHV-68 was inactivated using UV irradiation [25].

### Ethics statement

The animal care and use protocols were approved by Animal Care and Use Committee of Zhejiang University (Approval Number: 20160411). The policies of the State Scientific and Technological Commission of Regulations on the Administration of Laboratory Animals, Instructive Notions with Respect to Caring for Laboratory Animals issued by Ministry of Science and Technology of the People’s Republic of China, and Zhejiang Provincial Experimental Animal Management Measures were adhered.

### Cell stimulation and infection

BMMs, Raw264.7, MEF, NIH3T3, and MLE-12 cells were pre-treated with recombinant mouse IFN-γ at 10 U/mL for 12 hours before being infected with MHV-68. A multiplicity of infection (MOI) of 10 was used for BMMs and Raw264.7 cells, and of 1 for MLE-12, MEF and NIH3T3 cells through the study [7,72], based on the fact that the infectivity of MHV-68 cell is lower in BMMs than in Vero cells. Cells were incubated with the viral inoculum for 1 hour with rocking every 15 minutes at 37°C. After 1 hour of incubation, cells were washed twice with medium and cultured with fresh medium with or without IFN-γ. At the indicated times post infection, RNA, DNA, or protein was extracted for further experiments, or the whole cell culture was harvested for use in a plaque assay after being freeze-thawed three times. For the extraction of protein for western blotting, BMMs and NIH3T3 cells were infected with MHV-68 for the indicated periods and incubated with IFN-γ at 10 U/mL for the last one hour of infection.

For the inhibition assays, BMM cells were pre-treated with U0126 (an inhibitor of ERK1/2, 5 μM), SP600125 (an inhibitor of SAPK/JNK, 5 μM), SB239063 (an inhibitor of p38 MAPK, 10 μM) and Bay11-7082 (an inhibitor of NF-κB, 2 μg/mL) for 30 min before cells were infected with MHV-68 or stimulated with poly(I:C). At the indicated hours post infection, RNA or protein was extracted. U0126, SP600125, SB239063, and Bay 11–7082 were purchased from Beyotime Institute of Biotechnology (Shanghai, China).

### Plaque assay

Viral titers were determined by a plaque assay on Vero cells. Serial dilutions of the virus stocks, or the whole cell culture, were incubated with Vero cells for 1 hour. After incubation, the cells were overlaid with 1% (wt/vol) methylcellulose (Sigma, St. Louis, MO, USA) containing 50% (vol/vol) 2 × DMEM and 10% (vol/vol) FBS. The culture was continued for a further 5 days before the overlay medium were removed and cells were fixed and stained with 2% (wt/vol) crystal violet in 20% (vol/vol) ethanol. Wells containing 30–100 plaques were counted and the virus titers were calculated [33].

### DNA extraction and real-time PCR

To measure the copy number of the viral genome, total DNA was extracted from cells using the General AllGen kit (CW Biotech, Beijing, China). PCR was performed on the extracted DNA using an iTaq Universal SYBR Green Supermix (Bio-Rad, Richmond, CA, USA) and a CFX96 Touch Real-Time PCR Detection System (Bio-Rad) according to the manufacturer's instructions. The primers used were located in the ORF6 region of the MHV-68 genome (the primer sequences used are provided in Table 1). For each reaction, 150 ng of DNA was analyzed in duplicate, and a standard curve was obtained by measuring 1 to 10^6^ copies of a bacterial artificial chromosome containing the MHV-68 genome on a background of 150 ng of uninfected splenocyte DNA [73].

**Table 1:** Primer sequences for target genes.

Gene	Forward primer	Reverse primer
β-Actin	GTATCCTGACCCTGAAGTACC	TGAAGGTCTCAAACATGATCT
ORF50	GATTCCCCTTCAGCCGATAAG	CAGACATTGTAGAAGTTCAGGTC
ORF6	TGCAGACTCTGAAGTGCTGACT	ACGCGACTAGCATGAGGAGAAT
Socs1	CTGCGGCTTCTATTGGGGAC	AAAAGGCAGTCGAAGGTCTCG
Socs3	TGCGCCTCAAGACCTTCAG	GCTCCAGTAGAATCCGCTCTC
Socs6	TGCGCCTCAAGACCTTCAG	CATCAGGCTCTCGCTTTTGGA
Cis	ATGGTCCTTTGCGTACAGGG	GGTCTAGCACCTTCGGTTCAT
Shp2	GAAACGGTCATTCAGCCACT	GCAGCCAAGGAGTCATCTT
Tlr2	CTCTTCAGCAAACGCTGTTCT	GGCGTCTCCCTCTATTGTATTG
Tlr3	GTGAGATACAACGTAGCTGACTG	TCCTGCATCCAAGATAGCAAGT
Tlr4	GCCTTTCAGGGAATTAAGCTCC	GATCAACCGATGGACGTGTAAA
Tlr7	ATGTGGACACGGAAGAGACAA	ACCATCGAAACCCAAAGACTC
Tlr9	ACAACTCTGACTTCGTCCACC	TCTGGGCTCAATGGTCATGTG

### Quantitative RT-PCR

Total RNA was extracted using Ultrapure RNA Kit (CW Biotech). First-strand cDNA was synthesized using 1 μg of total RNA (DNase-treated) with an iScript cDNA synthesis kit (Bio-Rad). Reverse transcriptase PCR was performed using a 2720 Thermal Cycler (Applied Biosystems, Foster City, CA, USA). For each sample, a reaction without reverse-transcriptase was carried out to serve as a control to exclude the existence of endogenous genomic DNA. qPCR was then performed with 1 μL of cDNA using the iTaq Universal SYBR Green Supermix (Bio-Rad) on the CFX96 Touch Real-Time PCR Detection System (Bio-Rad). The primers sequences used to detect Socs1, Socs3, Socs6, Cis, Shp2, ORF50 (RTA), Tlr2, -3, -4, -7, -9 and β-actin are shown in Table 1. β-actin was amplified as an endogenous reference gene. The relative expression level was calculated by the ^ΔΔ^Ct method using Bio-Rad CFX Manager (Bio-Rad) and expressed as fold change relative to the control.

### Western blotting

Cells were lysed in 1 × RIPA buffer (Cell Signaling Technology, Danvers, MA, USA) containing 1 mM phenylmethylsulfonyl fluoride (PMSF) and a protease inhibitor cocktail. Protein concentration was determined by the Bradford protein assay. Total protein (20–40 μg) was separated by SDS-PAGE and blotted onto PVDF membranes. The blots were probed with antibodies against different proteins including; phospho-STAT1 (Tyr701), STAT1, SOCS1, phospho-p38, p38, phospho-Erk1/2, Erk1/2, phospho-SAPK/JNK, SAPK/JNK, phospho-IκBα, IκBα, phospho-NF-κB p65, NF-κB p65, and β-actin (all from Cell Signaling Technology), or with antibodies recognizing TLR2 (90kDa, DF7002), TLR3 (99kDa, DF6415), TLR4 (100kDa, AF7017), TLR7 (121kDa, DF6173), TLR9 (117kDa, DF2970), and MyD88 (33kDa, DF6162) (all from Affinity Biosciences, Cincinnati, OH, USA). To detect MHV-68 protein expression, an antibody detecting the late lytic expressed virus protein ORF65 [74] was used. The membranes were then probed with the appropriate horseradish-peroxidase-conjugated secondary antibody (Lianke, Hangzhou, China) followed by signal detection signal using a FluorChem E System (Protein Simple, Santa Clara, CA, USA). Quantification of the western blot bands was performed by densitometry using Image J Software (S2 Fig). The density for each band was normalized to that of β-actin. The relative protein expression of each protein was compared to the control, which was assigned a value of 1.

### Cell transfection

BMMs were transfected with the appropriate specific small interfering RNA (siRNA, GenePharma, Shanghai, China) using the INTERFERin transfection reagent (Polyplus transfection, Illkirch, France) at a final concentration of 50 nM, according to the manufacturer’s instructions. Briefly, on day 8 after BMM extraction, cells were plated into 12-well plates (1–2 × 10^5^/well) or 6-well plates (4–6 × 10^5^/well), allowed to grow overnight, and were then transfected with the indicated amounts of siRNA. The sequences of the individual siRNAs were provided by GenePharma and are shown in Table 2. NIH3T3 cells were transiently transfected with a SOCS1 expression plasmid (Ruijie, Shanghai, China) or a control plasmid (empty pcDNA3.1 vector) using Lipofectamine 2000 (Invitrogen, Carlsbad, CA) according to the manufacturer’s instructions. Briefly, NIH3T3 cells were plated into 12-well plates (8 × 10^4^/well) or 6-well plates (3 × 10^5^/well) and allowed to grow overnight. Following this the cells were transfected with the indicated amounts of a SOCS1 expression plasmid or a control plasmid. After 48 hours, the cells were collected for further experimentation. A small portion of cells was retained for western blotting to validate the knock down or overexpression of the target gene (S3 Fig). The transfection efficiency was monitored by co-transfection with a GFP-expressing plasmid, and at least a 60% efficiency was achieved.

**Table 2:** siRNA sequences.

Gene		Sequence
Socs1	Sense	CUACCUGAGUUCCUUCCCCdTdT
	Antisense	GGGGAAGGAACUCAGGUAGdTdT
Tlr2	Sense	GGAGUCUCUGUCAUGUGAUdTdT
	Antisense	AUCACAUGACAGAGACUCCdTdT
Tlr3	Sense	AUAACUUGCCAAUUGUCUGGAdTdT
	Antisense	UCCAGACAAUUGGCAAGUUAUdTdT
Tlr4	Sense	GUUCCAUUGCUUGGCGAAUGUdTdT
	Antisense	ACAUUCGCCAAGCAAUGGAACdTdT
Tlr7	Sense	GGGCAGACCUUAGACUUAAdTdT
	Antisense	UUAAGUCUAAGGUCUGCCCdTdT
Tlr9	Sense	UCCCUGUAUAGAAUGUGGCdTdT
	Antisense	GCCACAUUCUAUACAGGGAdTdT
Myd88	Sense	CAACCUGGGUCAAGUGUAAdTdT
	Antisense	UUACACUUGACCCAGGUUGdTdT
Control	Sense	UUCUCCGAACGUGUCACGUTTdTdT
	Antisense	ACGUGACACGUUCGGAGAAdTdT

### Statistical analysis

Statistical analysis was performed using GraphPad Prism software. Data are presented as mean ± standard errors of the means (S.E.M.). Statistical differences between two groups were assessed using Student’s *t*-test with significance set at *p* < 0.05.

## Supporting information

S1 Fig Mock-infected cells or cells infected with UV-inactivated MHV-68 showed sustained pSTAT1 expressions. In an experimental setting similar to that described for Fig 1C, BMMs were infected with MHV-68, UV-inactivated MHV-68, or mock-prepared viruses for the indicated periods of time. At the beginning of the last hour in each time period, 10 U/mL of IFN-γ (w/IFN-γ) or medium (w/o IFN-γ) was added to the culture medium as indicated. At the end of each period, the cells were collected for protein extraction, and western blotting was performed to evaluate the expression levels of STAT1 tyrosine phosphorylation (p-STAT1) and STAT1. At the same times, ORF65 western blotting was performed to measure MHV-68 protein expression levels.
(TIF)

S2 Fig Quantification of the western blot bands of this study. Quantification of the western blot bands was performed by densitometry using Image J Software. The density for each band was normalized to that of β-actin. The relative protein expression of each protein was compared to the control, which was assigned a value of 1. (A) Quantification of SOCS1 and TLR2, -3, -4, -7, -9 protein in Western blot of Fig 6A. (B) Quantification of SOS1 and TLR3 protein in Western blot of Fig 6C. (C) Quantification of SOCS1 and MyD88 protein in Western blot of Fig 6D. (D) Quantification of SOCS1 and protein in Western blot of Fig 7C.
(TIF)

S3 Fig The knock down or overexpression of SOCS1 in target cells. For each knock down or overexpression experiment targeting SCOS1, a portion of the cells without IFN-γ treatment was collected for SOCS1 western blotting. (A) Western blot analysis of SOCS1 protein in Fig 3A. (B) Western blot analysis of SOCS1 protein in Fig 3C. (C) Western blot analysis of SOCS1 protein in Fig 5A. (D) Western blot analysis of SOCS1 protein in Fig 5C.
(TIF)
