# Supplementary figures and images for: Inhibition of murine herpesvirus-68 replication by IFN-gamma in macrophages is counteracted by the induction of SOCS1 expression
Source: PLoS Pathog. 2018 Aug 3;14(8):e1007202. doi: 10.1371/journal.ppat.1007202 (PMC6093694; doi:10.1371/journal.ppat.1007202)

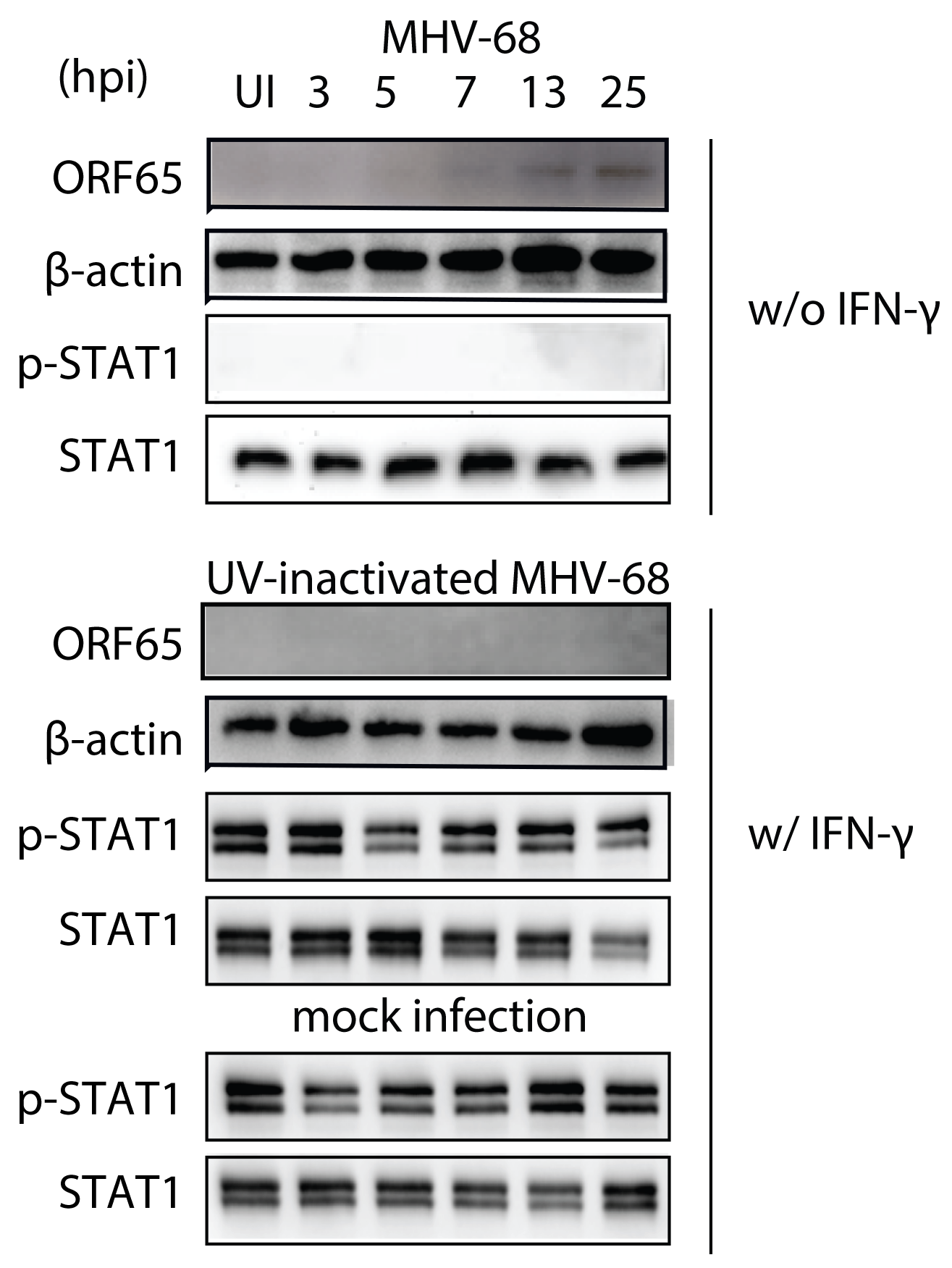

Supplement: S1 Fig — In an experimental setting similar to that described for Fig 1C, BMMs were infected with MHV-68, UV-inactivated MHV-68, or mock-prepared viruses for the indicated periods of time. At the beginning of the last hour in each time period, 10 U/mL of IFN-γ (w/IFN-γ) or medium (w/o IFN-γ) was added to the culture medium as indicated. At the end of each period, the cells were collected for protein extraction, and western blotting was performed to evaluate the expression levels of STAT1 tyrosine phosphorylation (p-STAT1) and STAT1. At the same times, ORF65 western blotting was performed to measure MHV-68 protein expression levels. (TIF) [file ppat.1007202.s001.tif]

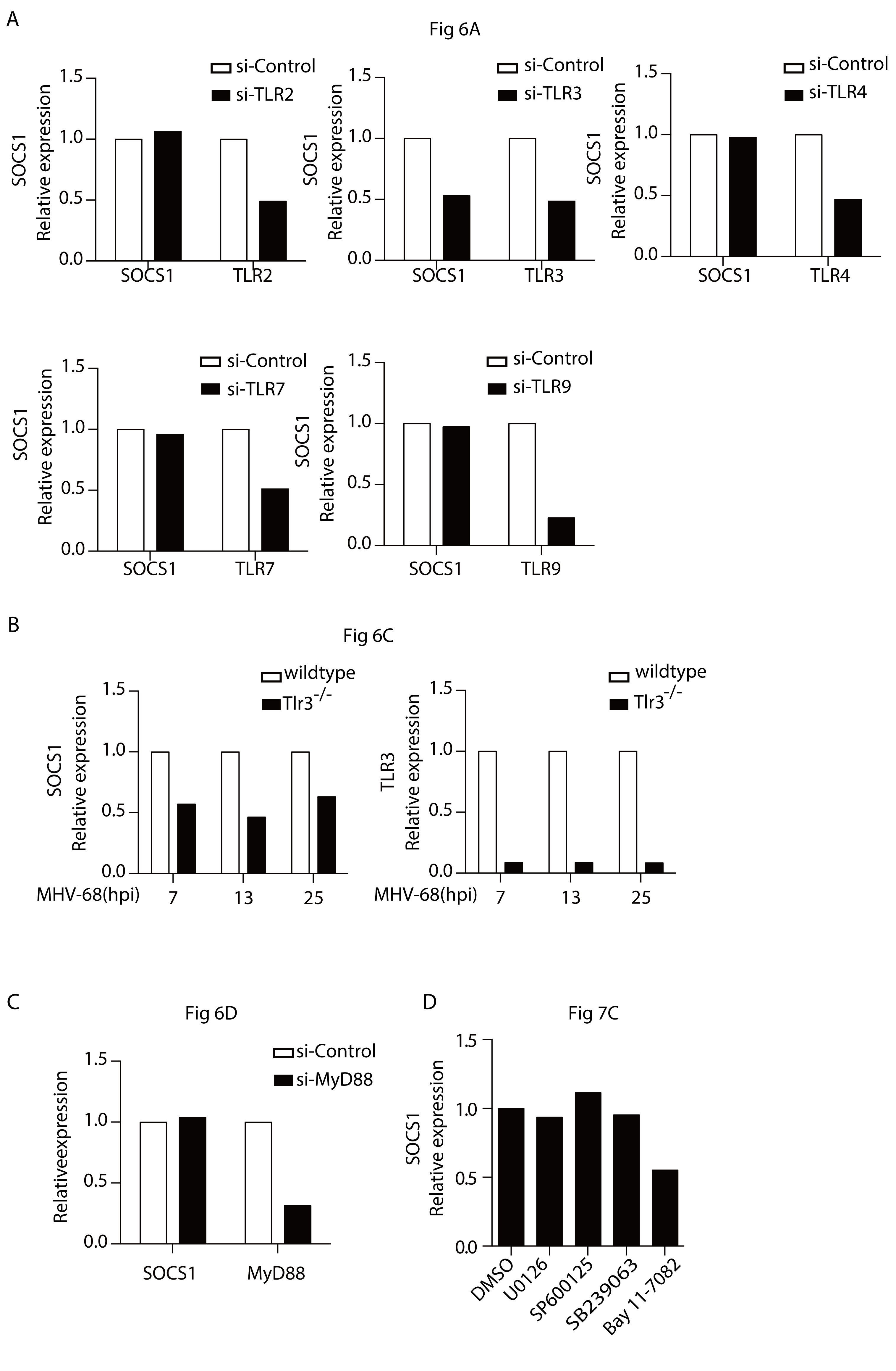

Supplement: S2 Fig — Quantification of the western blot bands was performed by densitometry using Image J Software. The density for each band was normalized to that of β-actin. The relative protein expression of each protein was compared to the control, which was assigned a value of 1. (A) Quantification of SOCS1 and TLR2, -3, -4, -7, -9 protein in Western blot of Fig 6A. (B) Quantification of SOS1 and TLR3 protein in Western blot of Fig 6C. (C) Quantification of SOCS1 and MyD88 protein in Western blot of Fig 6D. (D) Quantification of SOCS1 and protein in Western blot of Fig 7C. (TIF) [file ppat.1007202.s002.tif]

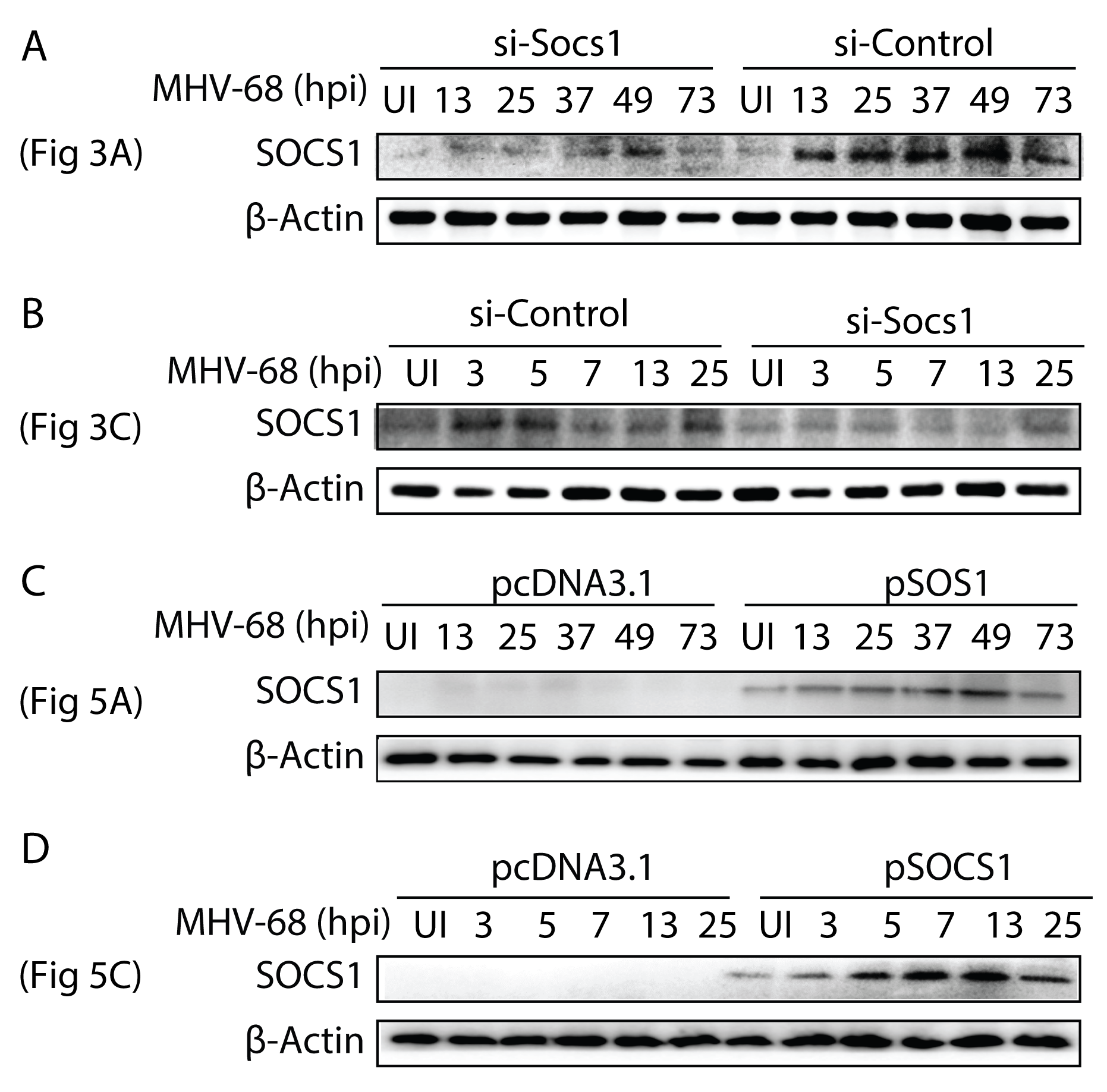

Supplement: S3 Fig — For each knock down or overexpression experiment targeting SCOS1, a portion of the cells without IFN-γ treatment was collected for SOCS1 western blotting. (A) Western blot analysis of SOCS1 protein in Fig 3A. (B) Western blot analysis of SOCS1 protein in Fig 3C. (C) Western blot analysis of SOCS1 protein in Fig 5A. (D) Western blot analysis of SOCS1 protein in Fig 5C. (TIF) [file ppat.1007202.s003.tif]
